# Distributive shock in cardiac intensive care unit patients

**DOI:** 10.1016/j.ihj.2025.09.008

**Published:** 2025-09-30

**Authors:** Sarvesh Pal Singh

**Affiliations:** Department of Cardio-Thoracic and Vascular Surgery, Cardio-Thoracic Sciences Center, All India Institute of Medical Sciences, New Delhi, 110029, India

## Abstract

Shock is a life-threatening condition of circulatory failure. Mixed shock is observed in approximately 24.5 % of patients with cardiogenic shock. In cases of out-of-hospital cardiac arrest, outcomes are worse when the predominant shock type is distributive rather than cardiogenic. Thus, distinguishing between cardiogenic and distributive phenotypes is crucial for appropriate management. A dysregulated immune response has been observed in both. This review article details the pathophysiology, diagnostic challenges, and management of distributive shock when encountered in a cardiac intensive care unit.

## 1 Introduction

Shock is a life-threatening condition of circulatory failure. It is a state of cellular tissue hypoxia caused by inadequate oxygen delivery that is unable to meet cellular metabolic needs and oxygen consumption requirements.^1^

## 2 Types of shock^2^

Based on etiology, the shock may be divided into.

1. Hypovolemic

Circulatory failure due to loss of adequate circulatory volume. If the cause of hypovolemia is acute blood loss, it is known as hemorrhagic shock.

2. Cardiogenic

Persistent hypotension with systolic blood pressure of less than 90 mm Hg and cardiac index less than 1.8 l/min/m2 without support and less than 2.0–2.2 l/min/m2 with support, and LVEDP >18 mm Hg or RVEDP >15 mm Hg.

3. Distributive (Vasodilatory)

Hypotension is caused by relative hypovolemia, which is caused by the redistribution of intravascular volume. This redistribution results from a decrease in systemic vascular resistance or an increase in capillary permeability.

4. Obstructive

The cause of hypotension is obstruction of the great vessels, heart, and/or lungs.

5. Mixed

Mixed shock has components of both cardiogenic and distributive shock. It could be triggered by a cardiac event (e.g., acute myocardial infarction), a vascular event (e.g., severe sepsis), or a common event (e.g., cardiac arrest). When the trigger for both components is the same, it's called a primary mixed shock.

## 3 Distributive shock in the cardiac intensive care unit (CICU)

Patients in the CICU can present with any shock mentioned earlier.^3^ Those presenting with cardiogenic shock can later evolve into or be complicated by a distributive component. Mixed shock is observed in approximately 24.5 % of patients with cardiogenic shock.^4^ In cases of out-of-hospital cardiac arrest (OHCA), outcomes are worse when the predominant shock type is distributive rather than cardiogenic.^5^ Interestingly, in these patients, post-resuscitation left ventricular ejection fraction (LVEF) does not correlate with mean arterial pressure or vasopressor requirements. Nonetheless, neurocognitive outcomes tend to be poorer in patients with LVEF greater than 40 % compared to those with LVEF below 40 %, even after adjusting for confounding variables. This suggests that patients with relatively preserved LVEF may predominantly exhibit distributive shock. Notably, the volume of fluid administered during the first 24 h appears to have no significant impact on either survival or neurocognitive outcomes. Therefore, distinguishing between cardiogenic and distributive phenotypes is crucial for appropriate management.

## 4 Epidemiology and clinical context

Patients presenting with heart failure or cardiogenic shock may develop distributive shock due to organ failure (e.g., acute liver failure, pancreatitis, adrenal insufficiency), sepsis, systemic inflammatory response syndrome (massive blood transfusion, post-CPR, etc.), drug-induced (nitroglycerine, calcium channel blockers, etc.), neurological events, and mechanical circulatory support.^4^, ^5^, ^6^, ^7^ Often seen as a sequela of cardiogenic shock, distributive shock may coexist from the beginning. A dysregulated immune response has been observed in both.^8^ In the former, inflammatory cytokines cause increased capillary permeability, decreased systemic vascular resistance, and arteriolar dilatation, whereas in the latter, the upregulation of inflammation causes myocardial dysfunction. Sepsis-induced myocardial dysfunction can occur in 20–65 % of adult septic shock patients.^9^ A recent study showed that patients requiring PCI and developing septic shock increased from 15 % to 24 % in 5 years (2016–2020).^10^

## 5 Pathophysiological insights

### 5.1 Distribution of adrenergic receptors (Table 1)

Adrenergic receptors are G protein-coupled, aminergic receptors with an intracellular carboxy and an extracellular amino terminus. The monoamines epinephrine and norepinephrine act as agonists on these receptors. Upon stimulation, the G proteins generate intracellular second messengers that cause the phosphorylation of G protein-coupled kinases, the release of intracellular calcium, the activation of ion pumps, and the modulation of gene expression. Due to various regulatory pathways and the widespread expression of G protein-coupled receptors, epinephrine and norepinephrine regulate cardiac output, vasoconstriction, vasodilation, metabolism, and cell proliferation.^11^ The adrenergic receptors are primarily of two types: alpha and beta. They are further subdivided into α1 (α1A, α1B andα1D), α2 (α2A, α2B and α2C), B1, B2 and B3 receptors.^12^

**Table 1:** Adrenergic receptors – types and locations.

S No	Type	Physiological response	Distribution
1	Alpha 1	mediate vasoconstriction via IP_3_-mediated calcium release	Vascular smooth muscle cells (SMCs), endothelial cells, cardiomyocytes, prostate SMCs, and brain.
2	Alpha 2	Inhibit adenylate cyclase, reducing cAMP and modulating sympathetic outflow	Autonomic ganglia, sympathetic nervous system, central nervous system, pancreas, platelets, kidney's tubular epithelium, vascular SMCs, and gastrointestinal SMCs.
3	Beta 1	predominantly affect the heart, enhancing inotropy and chronotropy	Cardiomyocytes, kidney SMCs, and adipocytes
4	Beta 2	mediate vasodilation and bronchodilation via increased cAMP	Vascular SMCs, endothelial cells, gastrointestinal SMCs, lung SMCs, cardiomyocytes, uterus SMCs, bladder SMCs, adipocytes, pancreas, eyes, liver, and skeletal muscle.
5	Beta 3	linked to lipolysis in adipose tissue	Cardiomyocytes, endothelial cells, adipocytes, brown adipocytes, bladder SMCs, gall bladder, retina, and epithelial cells.

During shock, stimulation of alpha-adrenergic receptors induces vasoconstriction, thereby increasing the effective circulating blood volume. Simultaneously, activation of the renin–angiotensin–aldosterone (RAA) axis further augments intravascular volume, while beta-adrenergic receptor stimulation enhances cardiac output through increased inotropy and chronotropy**.**^13^ The primary objective of these compensatory mechanisms is to preserve oxygen delivery to the brain and heart at the expense of less critical organs such as the kidneys and splanchnic circulation.^14^

The sympathetic nervous system primarily drives macrocirculatory regulation, whereas microcirculatory control is exerted locally via modulation of vascular tone. If compensation proves inadequate and dysoxia persists or worsens, hypoxic regions develop endothelial injury, heightened vascular tone, and increased capillary permeability. These changes herald the transition to the decompensatory phase, marked by local release of endothelin-1 and a subsequent reduction in cerebral perfusion.^15^

At this stage, shock-induced signalling promotes vascular smooth muscle relaxation, leading to vasodilation that counteracts the earlier neurohormonal vasoconstrictive efforts.^16^^,^^17^

In distributive shock, elevated levels of inflammatory cytokines and nitric oxide contribute to the downregulation of adrenergic receptors, resulting in a diminished cardiovascular compensatory response. Even after the return of spontaneous circulation (ROSC), systemic inflammation can persist for several days due to the continued release of damage-associated molecular patterns (DAMPs) and pathogen-associated molecular patterns (PAMPs). This sustained inflammatory state ultimately contributes to progressive end-organ injury.^8^^,^^17^

Zweck et al employed a machine learning algorithm—k-means clustering—to identify three distinct phenotypes in patients with cardiogenic shock, characterized by similar hemodynamic presentations: noncongested (Type I), cardiorenal (Type II), and cardiometabolic (Type III).^7^

• Patients in the noncongested group (Type I) were typically younger, without comorbidities, and with normal renal function.
• Those in the cardiorenal phenotype (Type II) tended to be older and presented with chronic kidney disease, diabetes mellitus, hypertension, and anemia.
• The cardiometabolic phenotype (Type III) was characterized by elevated lactate and transaminase levels, right ventricular failure, and tachycardia.

Patients in Type III were more likely to exhibit features of distributive shock—such as venous congestion, tissue dysoxia, and increased capillary permeability—arising from multi-organ injury (e.g., liver and kidney), systemic inflammation, and interleukin-mediated microcirculatory dysfunction.^7^ This systemic inflammatory response may also trigger neurohumoral activation, leading to increased release of natriuretic peptides.^18^

Therefore, in patients with predominant cardiogenic shock, the host response may be significantly modulated by the degree of systemic inflammation, the underlying phenotype, and intrinsic sources of heterogeneity, such as genetic alterations. Clonal hematopoiesis, defined as the expansion of a single hematopoietic stem cell harbouring a somatic mutation, has emerged as one such factor. Notably, its presence in patients with cardiogenic shock has been associated with increased mortality, suggesting a potential link between hematopoietic dysregulation and adverse inflammatory or immune responses in the setting of acute circulatory failure.^19^

## 6 Diagnostic challenges (Table 2

Both distributive shock and cardiogenic shock can manifest with hypotension, tachycardia, and altered mental status, though the latter is less likely in cardiogenic shock. Warm extremities in early distributive shock may mislead physicians away from the diagnosis of shock. Similarly, the absence of pulmonary edema in the early stages of cardiogenic shock may have the same effect. The inflammatory markers, lactate and troponin I, may be elevated in both conditions. An electrocardiogram may show ischemic changes or arrhythmias in cardiogenic shock, whereas it will be normal in distributive shock. Echocardiography in the early stages of both types may be indistinguishable due to hyperdynamic heart in distributive shock and compensatory mechanisms in cardiogenic shock. While point-of-care ultrasound (POCUS) shows high specificity for diagnosing both types, its sensitivity is low.

**Table 2:** Diagnostic challenges in differentiating cardiogenic vs distributive shock.

S. No	Tool	Cardiogenic Shock	Distributive shock
**1**	ECG	Ischemia/arrhythmias	Often normal
**2**	Echocardiography	Decreased ventricular function	Hyperdynamic LV
**3**	Lactate/Troponin I	Elevated	May be mildly elevated or normal
**4**	Hemodynamics	Increased PCWP, decreased cardiac index	Decreased SVR, increased cardiac index
**5**	Lung USG	B lines	Normal lung

A pulmonary artery catheter inserted early may help in the monitoring and add to the diagnostic value. An increased PCWP and decreased CI are suggestive of cardiogenic shock, whereas a markedly decreased SVR and increased CI point towards distributive shock. Mixed shock may have features of both types in various proportions.

## 7 Therapeutic options

The mainstay of treatment remains treating the underlying cause of distributive shock—sepsis, adrenal insufficiency, or neurological vasomotor failure. The initial supportive treatment involves fluid resuscitation, vasopressors, and inotropes. Often, the hemodynamic response to initial fluid boluses is interpreted as hypovolemia. However, volume responsiveness does not necessarily mean volume deficit. Due to increased vascular permeability and arteriolar dilatation, the excess fluid may shift to the third space, and the hemodynamic improvement may be short-lived. A conservative fluid strategy has been associated with lower costs, shorter lengths of stay, and durations of mechanical ventilation.^7^ Venous congestion has also been established as a risk factor for acute kidney injury (AKI) in shock.^20^

Maintaining an optimal fluid balance and knowing when to stop resuscitation are the most difficult challenges when managing shock. All the current invasive (Central venous catheter, PA catheter, Δ PCO2 between venous and arterial blood), semi-invasive (pulse contour-based cardiac output monitors), and noninvasive (Echocardiography, IVC diameter variability, Venous excess ultrasound grading) monitors are not without demerits. The most commonly used monitoring is central venous pressure, but changes in CVP cannot predict a hemodynamic response during a fluid challenge.^21^ The pulmonary artery catheter went out of critical care practice two decades ago for lack of survival benefits and increased adverse events.^22^, ^23^, ^24^ As the complexity of patients being managed in the ICU increases, the PAC may see a resurgence in managing patients with mixed shock, especially where the right ventricle is involved (cardiometabolic shock). A recent study by Sato et al concluded that patients with sepsis-induced cardiogenic shock may benefit from early (within 2 days of presentation) use of PAC compared to patients with sepsis alone, mainly when found to have echocardiography-documented cardiac dysfunction.^25^

Norepinephrine (NE) is the first-line vasopressor for treating distributive shock (Septic shock) due to its predominant alpha agonist properties and stimulation of B1 receptors.^26^ It causes intense vasoconstriction with mild tachycardia (and improves cardiac output). Doses up to 0.5 mic/kg/min have been used, but the commonly used dose is between 0.03 and 0.2 mic/kg/min.

Vasopressin, as a sole agent or in combination with NE (added before or after NE), has not yet been conclusively proven to decrease mortality in patients with distributive shock.^27^, ^28^, ^29^, ^30^ However, it may be associated with early shock reversal, a reduced incidence of renal failure, and a lower need for renal replacement therapy.^29^ Compared to NE, vasopressin in post-cardiac surgery patients was associated with a lower incidence of major complications, including atrial fibrillation, but no difference in mortality (24 of 151 in the NE group vs. 23 of 149 in the vasopressin group; adjusted OR 1.11; *p*-value 0.73).^28^ In septic shock patients, initiating vasopressin when the NE dose was less than 15 mic/min decreased 28 and 90-day mortality.^28^ A typical dose is 0.01–0.04 U/min or 0.6–2.4 U/h. Comparing cardiogenic shock with septic shock patients, vasopressin levels were found to be significantly lower (22.7 ± 2.2 pg/mL vs. 3.1 ± 1.0 pg/mL) in septic shock patients, and thus, a quick response to exogenously administered vasopressin.^31^

Methylene blue has been used to reverse vasoplegia in patients with distributive shock unresponsive to norepinephrine, vasopressin, or both without any survival benefit.^32^^,^^33^ One definition used for NE refractory vasoplegia is MAP <60 mm Hg, CI ≥ 2.5 L/min/m2, CVP <8 mm Hg, PCWP <10 mm Hg, and SVR <600 dyne s.cm-5 at NE dose of 0.5mic/kg/min.^33^ In the endothelium, it inhibits nitric oxide synthase (NOS) and decreases NO production. In the vascular smooth muscle cell, methylene blue inhibits soluble guanyl cyclase, preventing increased cGMP production inside the cell. Both these actions culminate in vasoconstriction. There is nonuniformity in dosing and duration of use for methylene blue. The dose of MB varies from 1.2 mg/kg to 3 mg/kg with or without infusion between 0.25 and 2.0 mg/kg/h for 4–12 h.^32^^,^^33^ A study performed in post-cardiac surgery patients with NE refractory vasoplegia concluded that after 48 h, there is no significant difference in SVR between groups with or without methylene blue. The commonest side effect of MB is greenish-blue colored urine, which is self-limiting. Rare side effects are hemolytic anemia in G-6 PD deficient patients and serotonin syndrome in patients receiving SSRIs, and only one case has been reported of pulmonary hypertensive crisis.^33^

Angiotensin II is the third-line vasopressor approved for distributive shock that does not stimulate the heart.^34^ Angiotensin-converting enzyme converts Angiotensin I to Angiotensin II. Endogenous angiotensin II levels increase in response to hypovolemia, activate sympathetic neurons, and cause an intracellular calcium increase, leading to vasoconstriction.^34^, ^35^, ^36^ Angiotensin II also increases the release of aldosterone, cortisol, and vasopressin, leading to sodium and water reabsorption and vasoconstriction.^34^^,^^36^^,^^37^ Early initiation of Angiotensin II in shock patients (those receiving ≤0.5 mic/kg/min of NE) is associated with a better increase in MAP and a decrease in the dose of NE.^37^ Similar to vasopressin, distributive shock patients with lower angiotensin II levels respond faster to exogenous AT II compared to those having higher levels.^38^ Although there is a theoretical possibility of an increased risk of thromboembolic events (increased thrombin formation and decreased thrombolysis), there has been no statistically significant difference in venous and arterial thromboembolism, valve thrombosis, or clots in mechanical circulatory support devices.^39^

Ventriculoarterial uncoupling (VAC) is a phenomenon observed in septic (distributive) shock.^40^ Systolic heart function is measured by end-systolic elastance (Ees), while the compliance of the arterial system is represented by arterial elastance (Ea). The ratio of Ea to Ees is known as the VAC Index, which reflects the efficiency of the heart and vascular system working together. An optimal VAC Index ranges from 0.5 to 1.2; values above 1.3 indicate uncoupling.^40^

Dobutamine can improve outcomes in distributive shock (especially in patients also receiving norepinephrine or vasopressin) by increasing stroke volume and Ees, which lowers the VAC Index by up to 35 %. However, if Dobutamine causes excessive tachycardia, it may increase Ea and counteract this benefit. When various combinations of inotropes were studied for mixed shock (distributive with cardiogenic shock), the Norepinephrine–Dobutamine combination was associated with the lowest 28-day all-cause mortality.^41^

Often, patients with mixed shock or distributive shock with or without NE (±dobutamine) demonstrate tachycardia. This tachycardia may be adaptive or maladaptive. Several studies have shown the beneficial effect of Esmolol (β-blockers) on hemodynamics and mortality in patients with septic shock.^42^, ^43^, ^44^, ^45^ This beneficial effect might arise from treating maladaptive tachycardia, which may cause or sustain the shock. In maladaptive tachycardia, there is an increase in end-systolic pressure (ESP) and, therefore, Ea (=ESP/SV). Esmolol decreases the Ea and improves stroke volume and VAC index in patients with preserved LVEF.^46^

Evidence is mounting regarding the heterogeneity of the host response to shock and its treatment.^44^ The activation of the sympathetic system in shock is independently influenced by the endothelial phenotype (increased expression of bio-adrenomedullin or angiopoietin 2), irrespective of the type of shock.^47^, ^48^, ^49^, ^50^, ^51^ Similarly, the early response to any shock involves a common pattern involving signaling molecules such as DAMPs, TLR4, and alarmins.^52^ However, patients' responses to a standard treatment protocol may differ even with overlapping gene and protein expression in different types of shock. The unpredictable response to vasopressors and inotropes in septic shock is related to the patient's immune phenotype, specifically the sepsis response signature (SRS1 or SRS2).^53^ Commonly, there is an increased inflammatory response to infection (SRS 2), but immunosuppression may also occur in some patients (SRS 1) and is associated with increased mortality.^53^

## 8 Conclusion

Identifying the phenotype of distributive predominant shock in cardiac patients and early use of vasopressors decreases major complications and all-cause mortality in the CICU. Host response to shock management is heterogeneous.

## Declaration of competing interest

The authors declare that they have no known competing financial interests or personal relationships that could have appeared to influence the work reported in this paper.
